# TMEM161B‐AS1 suppresses proliferation, invasion and glycolysis by targeting miR‐23a‐3p/HIF1AN signal axis in oesophageal squamous cell carcinoma

**DOI:** 10.1111/jcmm.16652

**Published:** 2021-05-27

**Authors:** Zuxuan Shi, Guanghui Li, Zhen Li, Junhao Liu, Yu Tang

**Affiliations:** ^1^ Department of Medical Oncology Henan Provincial People’s Hospital People’s Hospital of Zhengzhou University Zhengzhou China; ^2^ Department of Endocrinology Henan Provincial People’s Hospital Zhengzhou China; ^3^ Department of Endocrinology of Central China Fuwai Hospital Central China Fuwai Hospital of Zhengzhou University Zhengzhou China

**Keywords:** competitive endogenous RNA, long non‐coding RNA, oesophageal squamous cell carcinoma, prognosis, TMEM161B‐AS1

## Abstract

Mounting data have shown that long non‐coding RNAs (lncRNAs) widely participate in tumour initiation, development, progression and glycolysis in a variety of tumours. However, the clinical prognosis and molecular mechanisms of TMEM161B‐AS1 in oesophageal squamous cell carcinoma (ESCC) remain still unknown. Here, TMEM161B‐AS1 and HIF1AN were significantly lower in ESCC tissues than in normal samples, and their low expressions were both related to TNM stage, lymph node metastasis and poor prognosis of ESCC patients. Functionally, TMEM161B‐AS1 overexpression or miR‐23a‐3p depletion suppressed the proliferation, invasion and glycolysis as well as reduced glucose consumption and lactate production in ESCC cells. Mechanistically, TMEM161B‐AS1 manipulated HIF1AN expression by competitively sponging miR‐23a‐3p in ESCC cells. MiR‐23a‐3p mimic and HIF1AN siRNA partly reversed cell phenotypes mediated by TMEM161B‐AS1 in ESCC cells. Collectively, TMEM161B‐AS1, miR‐23a‐3p and HIF1AN may be tightly involved in ESCC development and progression as well as patients’ prognosis, and TMEM161B‐AS1/miR‐23a‐3p/HIF1AN signal axis may be a promising target for the treatment of ESCC patients.

## 1 INTRODUCTION

Oesophageal cancer (ESCA) is the sixth worst prognosis and the eighth most frequent occurring tumour with high aggressiveness and poor survival in the world.^1^, ^2^, ^3^, ^4^ The two main histological subtypes of ESCA are oesophageal adenocarcinoma (EAC) and oesophageal squamous cell carcinoma (ESCC),^5^ and ESCC is still the predominant histological type of ESCA worldwide, accounting for more than 80% of all ESCA cases.^6^ ESCC is highly prevailing in Asia, especially in Henan Province, China.^7^, ^8^ Although a great number of therapeutic strategies have been developed for ESCA patients, the overall five‐year survival rate is still far from satisfactory.^9^, ^10^ Thus, considering the high morbidity and mortality of ESCA, it is badly in need to seek for new biomarkers for early diagnosis and prognostic determination as well as therapeutic strategies for ESCA patients.

Recently, long non‐coding RNAs (lncRNAs) have been identified to be involved in tumorigenesis.^11^ LncRNAs as a class of non‐coding RNAs are more than 200 nucleotides in length, but be lack of protein‐coding probability, and are widely implicated in regulation of gene expression at three different levels, including transcriptional level, post‐transcriptional level and epigenetic level.^12^, ^13^ Functionally, lncRNAs manipulated multiple different complex biological processes by various molecular mechanisms, such as sponging of microRNAs (miRNAs), alternative splicing, recruitment of chromatin‐related proteins and the regulation of gene transcription.^14^, ^15^, ^16^, ^17^ Clinically, many lncRNAs have been shown to be underlying diagnostic and prognostic biomarkers in a majority of different type tumours.^18^, ^19^, ^20^, ^21^ Metabolically, many lncRNAs are considered to be important for tumour cells to survive and grow by altering glucose and lipid metabolisms.^22^, ^23^, ^24^, ^25^, ^26^ These data highlighted the biological significance and clinical value of lncRNAs in a variety of tumours, and thus, lncRNAs and its related regulatory pathways may be novel therapeutic strategies for tumour patients.

Currently, TMEM161B‐AS1 has been verified to be poor expression in endometrial cancer cell HEC‐50 derivatives exhibiting high invasive ability,^27^ implying its close association with tumour invasion. However, the functions and exact molecular mechanisms of TMEM161B‐AS1 in ESCC are not well defined. In this study, the TMEM161B‐AS1 expression patterns in ESCC tissues and cells were detected, and its biological functions in cell proliferation, invasion and glycolysis were confirmed by gain of function (GOF) and loss of function (LOF). Mechanistically, TMEM161B‐AS1 served as a molecular sponge by absorbing miR‐23a‐3p, a vital tumour oncogene that promotes ESCC proliferation, invasion and glycolysis by directly targeting HIF1AN and glycolysis‐related proteins. Collectively, our data highlighted the essential glycolysis‐related signal axis TMEM161B‐AS1/miR‐23a‐3p/HIF1AN that reprograms ESCC glucose metabolism, and thus, targeting this signal axis may be a promising strategy for the treatment of ESCC patients.

## 2 MATERIALS AND METHODS

### 2.1 Tissue samples and cell lines

ESCC tissues and paired normal samples were obtained from 63 cases of patients who underwent curative surgery, which was approved by the Research and Ethics Committee of Henan Provincial People's Hospital (No. 202131). All tissue samples were examined by H&E staining and were confirmed as ESCC by experienced pathologists. Informed consents of all ESCC samples were obtained from each patient in this study. Clinicopathological features were as follows: ≥60 years old, 44 cases, <60 years old, 19 cases; male, 41 cases, female, 22 cases; superficial layer, 27 cases, deep layer, 36 cases; high/medium differentiation, 32 cases, poor differentiation, 31 cases; TNM stage I + II, 28 cases, III+IV, 35 cases; lymph node metastasis, 26 cases; and without lymph node metastasis, 37 cases. Human ESCC cell lines including Eca109, KYSE30, KYSE70, KYSE150 and KYSE450 as well as Het‐1A (normal oesophageal epithelial cell) were purchased from the Chinese Academy of Sciences Cell Bank, and all cell lines above were cultured in RMPI 1640 medium harbouring 10% foetal bovine serum (Gibco, Invitrogen) in a humidified incubator harbouring 5% CO_2_.

### 2.2 Bioinformatics assay

TMEM161B‐AS1 and miR‐23a‐3p levels were investigated by StarBase v3.0 online tool, a web‐based tool for searching for expression of non‐coding RNA or coding RNA. The microarray expression GEO data set related to ESCC (GSE43732) was downloaded from the GEO database for investigation for miR‐23a‐3p expression. The coding probability of TMEM161B‐AS1 was predicted using online tool CPAT from the website http://lilab.research.bcm.edu/cpat/. The binding sites of miR‐23a‐3p in TMEM161B‐AS1 transcript were predicted using DIANA‐LncBase v2 online tool. TargetScan and miRDB were performed to predict the possible downstream target genes of miR‐23a‐3p.

### 2.3 Vector construction and cell transfection

TMEM161B‐AS1 siRNA#1, 2 and 3 were synthesized by Guangzhou RiboBio Co., Ltd. Si‐NC (negative control) was also purchased from Guangzhou RiboBio Co., Ltd. pcDNA3.1 and pcDNA3.1‐TMEM161B‐AS1 and pcDNA3.1‐HIF1AN were constructed by TSINGKE Biological Technology. HIF1AN siRNA was purchased from Santa Cruz Company. Mimics and inhibitors of miR‐23a‐3p and NC were all purchased from GenePharma Company (GenePharma). ESCC cells were transfected using Lipofectamine^TM^ 3000 (Invitrogen) according to the manufacturer's instruction. Briefly, a total of 5 × 10^5^ ESCC cells were seeded into each well in a 6‐well plate and transfected with siRNAs (100 nmol/L) or pcDNA3.1‐related plasmids (approximately 2μg) upon reaching about 80% confluence. The related experiments except for proliferation assay at indicated time‐points were determined 48 hours after transfection.

### 2.4 Real‐time quantitative PCR (qRT‐PCR)

For qRT‐PCR assay of HIF1AN and TMEM161B‐AS1 levels, total RNA was extracted by TRIzol reagent (Invitrogen) as described in the manufacturer's protocol. qRT‐PCR was carried out by Quant one step qRT‐PCR kit (SYBR Green, FP303) (Tiangen Biotech) on an ABI 7500 machine (Applied Biosystems) using the following primers (TMEM161B‐AS1 F: 5'‐AGCTGATGTGACGTGGCAAT‐3', R: 5'‐GGCAGCACAGTCTTTCATCC‐3', length: 213bp; HIF1AN F: 5'‐TGAGAATGAGGAGCCTGTGGT‐3', R 5'‐TGTTGGACCTCGGCTTAAAGT‐3', length: 191bp; GAPDH‐F: 5'‐GGGAGCCAAAAGGGTCATCA‐3'; GAPDH‐R 5'‐AGTGATGGCATGGACTGTGG‐3', length: 205bp). For miR‐23a‐3p assay, cDNA was obtained by the miRcute Plus miRNA First‐Strand cDNA Kit (Tiangen Biotech, Beijing). The miR‐23a‐3p level was investigated using the miRcute Plus miRNA qPCR Kit (SYBR Green) (Tiangen Biotech) using the following specific forward primers along with the reverse primers in the kit (miR‐23a‐3p F: 5'‐ATCACATTGCCAGGGATTTCC‐3'; U6‐F: 5'‐CTCGCTTCGGCAGCACA‐3'), and U6 was used as internal control.

### 2.5 Cell proliferation assay

Cell proliferation of Eca109 and KYSE30 cells was performed as described in the manufacturer's protocol, and the experiment was conducted in triplicate. Briefly, ESCC cells (2000 cells/well) were added to 96‐well plate. At 24, 48, 72 and 96 hours, the Cell Counting Kit‐8 (CCK‐8) (Beyotime Biotech) was applied to each experimental well, and absorbance value (450nm) was determined in a microplate reader (Thermo Scientific).

### 2.6 Cell invasion assay

The Transwell assay was employed to examine cell invasion of ESCC cells using Transwell chamber harbouring Matrigel (BD Biosciences). Briefly, Eca109 and KYSE30 cells (1 × 10^5^) were added to the upper layer of chamber, and the underlayer of chamber was covered by 20% FBS. Rinsing two times using PBS buffer, invasive cells were fixed using methanol (about 800 μL) for 20 minutes and then 0.1% of crystal violet was utilized to stain the invasive cells for 20 minutes 48 hours after transfection. Finally, the pictures were taken under the field of 200× magnification.

### 2.7 Glucose uptake and lactate production assays

Glucose assay kit (Shanghai Rongsheng Biotech Co., Ltd) and lactate assay kit (Nanjing Jiancheng Bioengineering Institute) were used to determine the glucose consumption and lactate production according to the manufacturer's instructions, respectively. All data obtained were normalized to protein quantitative values.

### 2.8 Subcellular fractionation

The nuclear RNA and cytoplasmic RNA were extracted by cell nucleus and cytoplasm RNA isolation kit (Beibei, Biotech, Co., Ltd) according to the manufacturer's instruction. Subsequently, qRT‐PCR was utilized to determine the gene expression using TMEM161B‐AS1, U6 and GAPDH specific primers.

### 2.9 Fluorescence in situ hybridization (FISH)

TMEM161B‐AS1 probe was synthesized and labelled using Cy3 by GenePharma Company. For FISH assay, Eca109 and KYSE30 cells were grown in 24‐well plates with glass coverslips for 24 hours. After immobilization and permeabilization, Eca109 and KYSE30 cells were hybridized with 20 μmol/L Cy3‐labelled TMEM161B‐AS1 probe, and 6‐diamidino‐2‐phenylindole (DAPI) was used to stain cell nuclei of ESCC cells. The images were observed with a florescent microscope.

### 2.10 Dual‐luciferase reporter assay

The interaction of miR‐23a‐3p with TMEM161B‐AS1 or HIF1AN was performed using the dual‐luciferase reporter assay system in Eca109 and KYSE30 cells. The plasmids pmirGLO‐TMEM161B‐AS1‐wild‐type (pmirGLO‐TMEM161B‐AS1‐WT) and pmirGLO‐TMEM161B‐AS1‐mutation (pmirGLO‐TMEM161B‐AS1‐MUT) as well as pmirGLO‐HIF1AN‐WT and pmirGLO‐HIF1AN‐MUT (TSINGKE Biological Technology) along with miR‐23a‐3p mimic and NC mimic were transfected into ESCC cells using Lipofectamine^TM^ 3000, respectively. The Dual‐Luciferase Reporter Assay System (Promega) was used to determine the luciferase activity 48h after transfection as described in the manufacturer's protocol.

### 2.11 RNA immunoprecipitation (RIP)

RNA‐binding protein immunoprecipitation kit was purchased from Millipore Company. RIP experiment was performed in ESCC cells according to the manufacturer's protocol. Briefly, RIP lysates were isolated from Eca109 and KYSE30 cells treated with miR‐23a‐3p mimic or NC mimic and then were applied to immunoprecipitation using either 5μl of anti‐Ago2 antibody or 5μl of a normal mouse IgG using RNA‐binding Protein Immunoprecipitation Kit. The TMEM161B‐AS1 and miR‐23a‐3p enriched on beads was determined by qRT‐PCR using corresponding specific primers.

### 2.12 Western blot

Total proteins were obtained using RIPA lysis (Solarbio) from ESCC cells, and Bradford method was utilized to determine the protein concentration. The separation of the proteins was performed using SDS‐PAGE and then was transferred to PVDF membrane (Millipore Corporation). After blocking with skimmed milk, the primary antibodies against HIF1AN (ab227550, 1:500), HIF‐1α (ab51608, 1:200), HK2 (ab227198, 1:5000), PFKM (ab154804, 1:1000), LDHA (ab101562, 1:1000) and β‐actin (ab8227, 1:1000) (Abcam) were applied to PVDF membrane (Roche, Switzerland) overnight at room temperature (RT). Then, the secondary antibody (ZSGB‐BIO) was incubated with PVDF membrane. Finally, the protein signal was developed using enhanced chemiluminescence (ECL) reagent (Beyotime).

### 2.13 Statistical assay

GraphPad Prism v8.0 software was performed to examine all experimental data. All data were expressed as mean ±standard deviation (SD). Spearman was used to investigate the non‐parametric data, and Pearson was performed to analyse the parametric data. Log‐rank test was used to determine the statistical difference of survival, and survival curves were drawn using Kaplan‐Meier. For the matched samples, the data were investigated using Wilcoxon signed rank, and for non‐matched samples, the data were compared by Mann‐Whitney test. The comparison between two groups was determined using a Student's *t* test, the comparison of ≥3 groups was determined using one‐way ANOVA, and then, Bonferroni test was selected for further statistical assay when data sets contain >3 groups. A *P* value less than .05 was considered to indicate a statistically significant difference.

## 3 RESULTS

### 3.1 Reduced TMEM161B‐AS1 and HIF1AN expressions in ESCC tissues and their low expressions predict poor prognosis of ESCC patients

**FIGURE 1:**
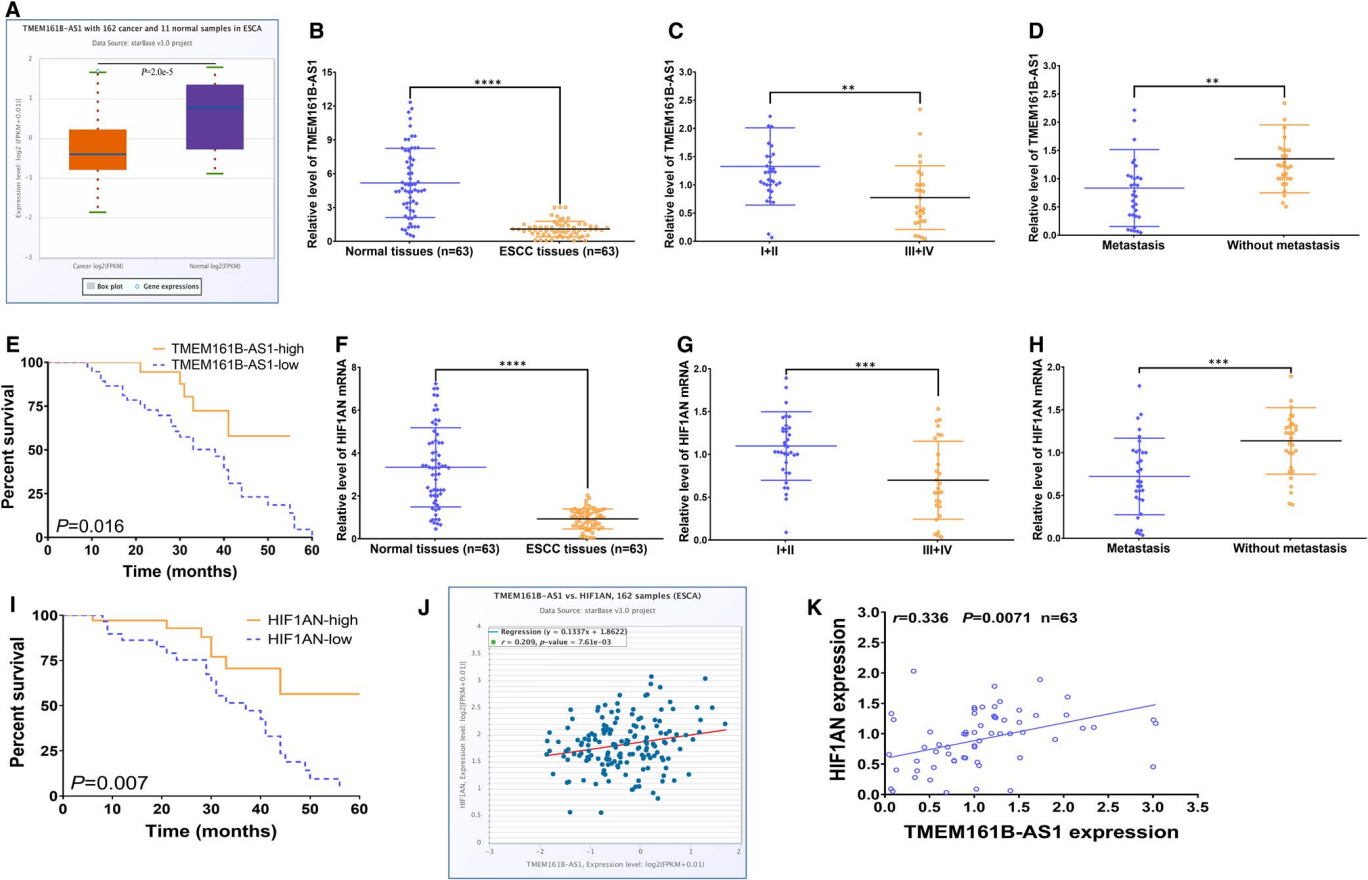
Reduced expressions of TMEM161B‐AS1 and HIF1AN in ESCC tissues and their associations with prognosis of ESCC patients. A, StarBase v3.0 online tool was performed to investigate the TMEM161B‐AS1 expression in 162 cases of ESCA samples and 11 normal samples. B, qRT‐PCR assay for the TMEM161B‐AS1 expression in 63 cases of ESCC samples and paired normal samples. C, The expression of TMEM161B‐AS1 in ESCC samples with I + II stage and III + IV stage. D, The expression of TMEM161B‐AS1 in ESCC samples with lymph node metastasis and without lymph node metastasis. E, Log‐rank test was used to determine the association of TMEM161B‐AS1 expression with the prognosis of ESCC patients. F, qRT‐PCR assay for the HIF1AN expression in 63 cases of ESCC samples and paired normal samples. G, The expression of HIF1AN in ESCC samples with I + II stage and III + IV stage. H, The expression of HIF1AN in ESCC samples with lymph node metastasis and without lymph node metastasis. I, Log‐rank test was used to determine the association of HIF1AN expression with the prognosis of ESCC patients. J, StarBase v3.0 online tool was carried out to examine the correlation of TMEM161B‐AS1 expression with HIF1AN expression in 162 cases of ESCA samples. K, Pearson correlation assay was performed to detect the correlation of TMEM161B‐AS1 expression with HIF1AN expression in 63 cases of ESCC tissues. **P < .01, ***P < .001 and ****P < .0001, indicating statistical significance

To investigate the expressions of TMEM161B‐AS1 and HIF1AN in ESCC tissues and their clinic values, TCGA database and qRT‐PCR were employed to examine their levels in ESCC tissues. TCGA database revealed that TMEM161B‐AS1 was significantly down‐regulated in ESCA tissues (162 cases) compared with normal samples (11 cases) (*P* < .0001) (Figure 1A). Further qRT‐PCR assay demonstrated that TMEM161B‐AS1 expression in 63 cases of ESCC samples was markedly lower than that in paired 63 cases of normal samples (*P* < .0001; Figure 1B). Furthermore, TMEM161B‐AS1 expression in ESCC patients with III and IV stage as well as lymph node metastasis was dramatically lower than that in ESCC patients with I and II stage as well as without lymph node metastasis (*P* < .01; Figure 1C,D). Importantly, ESCC patients with high TMEM161B‐AS1 expression displayed higher survival rate, compared with those with low TMEM161B‐AS1 expression (Figure 1E). Besides, HIF1AN displayed the similar expression pattern with TMEM161B‐AS1 in ESCC tissues and ESCC tissues with different TNM stage and differential metastatic status (Figure 1F‐H). Similarly, ESCC patients harbouring high HIF1AN expression exhibited the higher survival rate than those with low HIF1AN expression (Figure 1I). The data from TCGA and qRT‐PCR revealed that TMEM161B‐AS1 presented positive correlation with HIF1AN expression in ESCC samples (Figure 1J,K). These data suggest that TMEM161B‐AS1 and HIF1AN may participate in ESCC progression and may be a promising predictor for the prognosis of ESCC patients.

### 3.2 TMEM161B‐AS1 suppresses cell proliferation, invasion and glycolysis in ESCC cells

**FIGURE 2:**
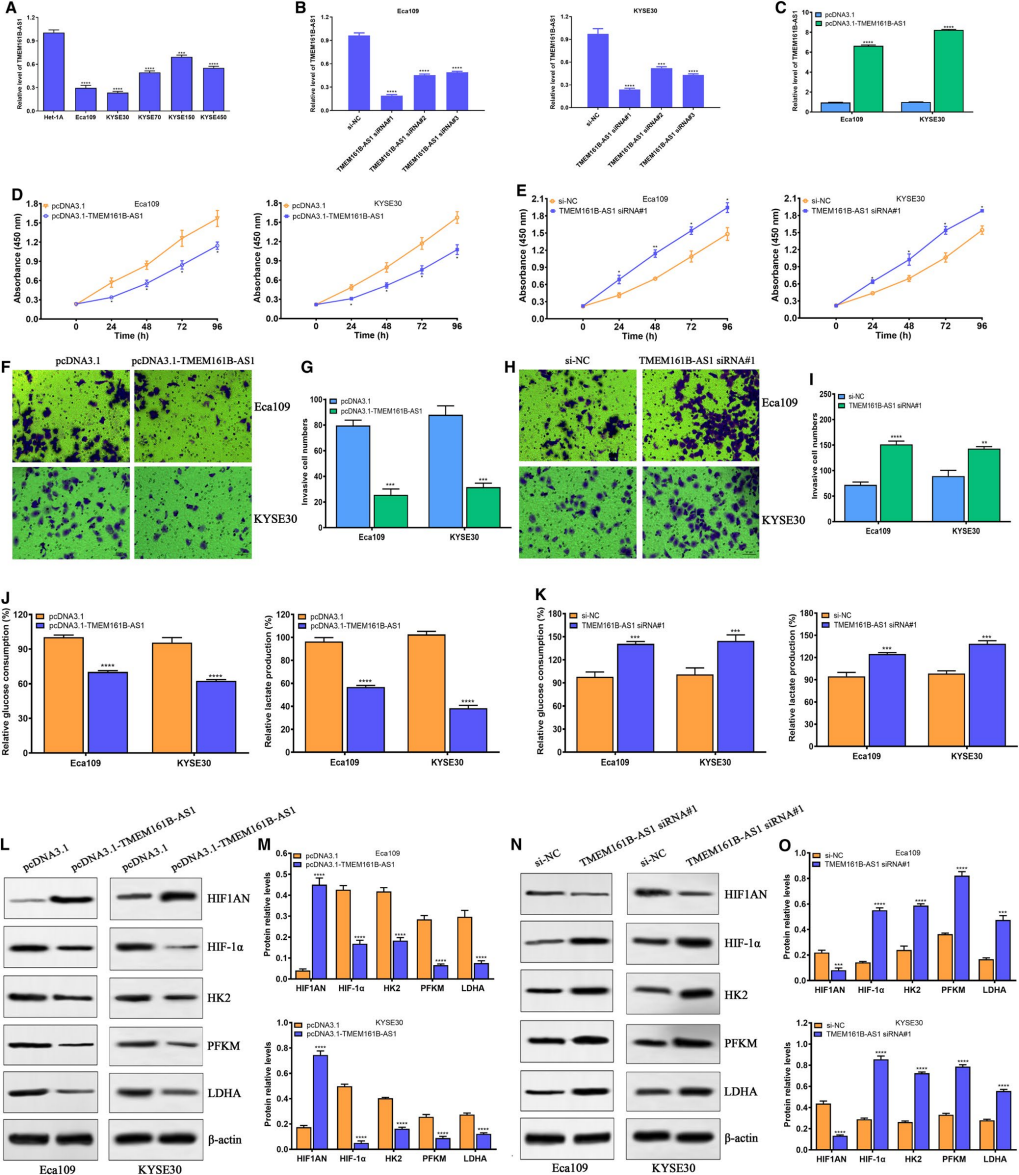
TMEM161B‐AS1 suppresses the proliferation, invasion and glycolysis of ESCC cells. A, qRT‐PCR was performed to investigate the expression of TMEM161B‐AS1 in a panel of ESCC cells (Eca109, KYSE30, KYSE70, KYSE150 and KYSE450) and normal oesophageal epithelial cell Het‐1A. B, Three siRNAs against TMEM161B‐AS1 significantly down‐regulated the expression of TMEM161B‐AS1 in Eca109 and KYSE30 cells at 48 h after transfection. C, pcDNA3.1‐TMEM161B‐AS1 promoted the expression of TMEM161B‐AS1 in Eca109 and KYSE30 cells at 48 h after transfection. D, TMEM161B‐AS1 overexpression significantly suppressed cell proliferation in Eca109 and KYSE30 cells at indicated time‐points. E, TMEM161B‐AS1 knockdown markedly promoted cell proliferation in Eca109 and KYSE30 cells at indicated time‐points. F and G, TMEM161B‐AS1 up‐regulation inhibited cell invasion in Eca109 and KYSE30 cells at 48 h after transfection, bar = 50 μm. H and I, TMEM161B‐AS1 depletion enhanced cell invasion in Eca109 and KYSE30 cells at 48 h after transfection, bar = 50 μm. J, TMEM161B‐AS1 overexpression suppressed glucose consumption and lactate production in Eca109 and KYSE30 cells at 48 h after transfection. K, TMEM161B‐AS1 knockdown promoted glucose consumption and lactate production in Eca109 and KYSE30 cells at 48 h after transfection. L and M, TMEM161B‐AS1 overexpression elevated HIF1AN expression, but reduced the expressions of HIF‐1α, HK2, PFKM and LDHA in Eca109 and KYSE30 cells at 48 h after transfection. N and O, TMEM161B‐AS1 depletion suppressed HIF1AN expression, but increased the expressions of HIF‐1α, HK2, PFKM and LDHA in Eca109 and KYSE30 cells at 48 h after transfection. *P < .05, **P < .01, ***P < .001 and ****P < .0001, indicating statistical significance

Given the essential role of TMEM161B‐AS1 in ESCC progression and metastasis, we next investigated the biological functions of TMEM161B‐AS1 in ESCC. Firstly, the endogenous expression of TMEM161B‐AS1 was determined in a panel of ESCC cell lines, and Eca109 and KYSE30 with relatively lower TMEM161B‐AS1 level were selected for further functional assays (Figure 2A). qRT‐PCR assay demonstrated that siRNAs against TMEM161B‐AS1 significantly down‐regulated the expression of TMEM161B‐AS1 in Eca109 and KYSE30 cells (Figure 2B), whereas pcDNA3.1‐TMEM161B‐AS1 markedly promoted the expression of TMEM161B‐AS1 in Eca109 and KYSE30 cells (Figure 2C). CCK‐8 experiment showed that TMEM161B‐AS1 overexpression extremely inhibited the proliferation of ESCC cells (Figure 2D), whereas TMEM161B‐AS1 depletion promoted the proliferation of ESCC cells (Figure 2E). Moreover, TMEM161B‐AS1 overexpression evidently suppressed cell invasion of Eca109 and KYSE30 cells (Figure 2F,G), whereas TMEM161B‐AS1 knockdown dramatically enhanced cell invasion of Eca109 and KYSE30 cells (Figure 2H,I). Collectively, these data imply that TMEM161B‐AS1 functions as tumour suppressor in ESCC.

Considering the correlation of TMEM161B‐AS1 function with HIF1AN, we further investigated the biological function of TMEM161B‐AS1 in ESCC glycolysis. We found that TMEM161B‐AS1 overexpression inhibited glucose consumption and lactate production in Eca109 and KYSE30 cells (Figure 2J), whereas TMEM161B‐AS1 down‐regulation promoted glucose consumption and lactate production in Eca109 and KYSE30 cells (Figure 2K). To further dissect the possible molecular mechanisms of TMEM161B‐AS1 implicated in ESCC glycolysis, we performed the Western blot assay for investigation of the expressions of glycolysis‐related proteins. We found that TMEM161B‐AS1 overexpression promoted the expression of HIF1AN, but suppressed the expressions of HIF‐1α, HK2, PFKM and LDHA in Eca109 and KYSE30 cells (Figure 2L,M), and converse data were obtained when TMEM161B‐AS1 was depleted in Eca109 and KYSE30 cells (Figure 2N,O). These findings suggest that TMEM161B‐AS1 plays a key regulatory role in ESCC glycolysis.

### 3.3 TMEM161B‐AS1 functions as competitive endogenous RNA (ceRNA) by absorbing miR‐23a‐3p in ESCC cells

**FIGURE 3:**
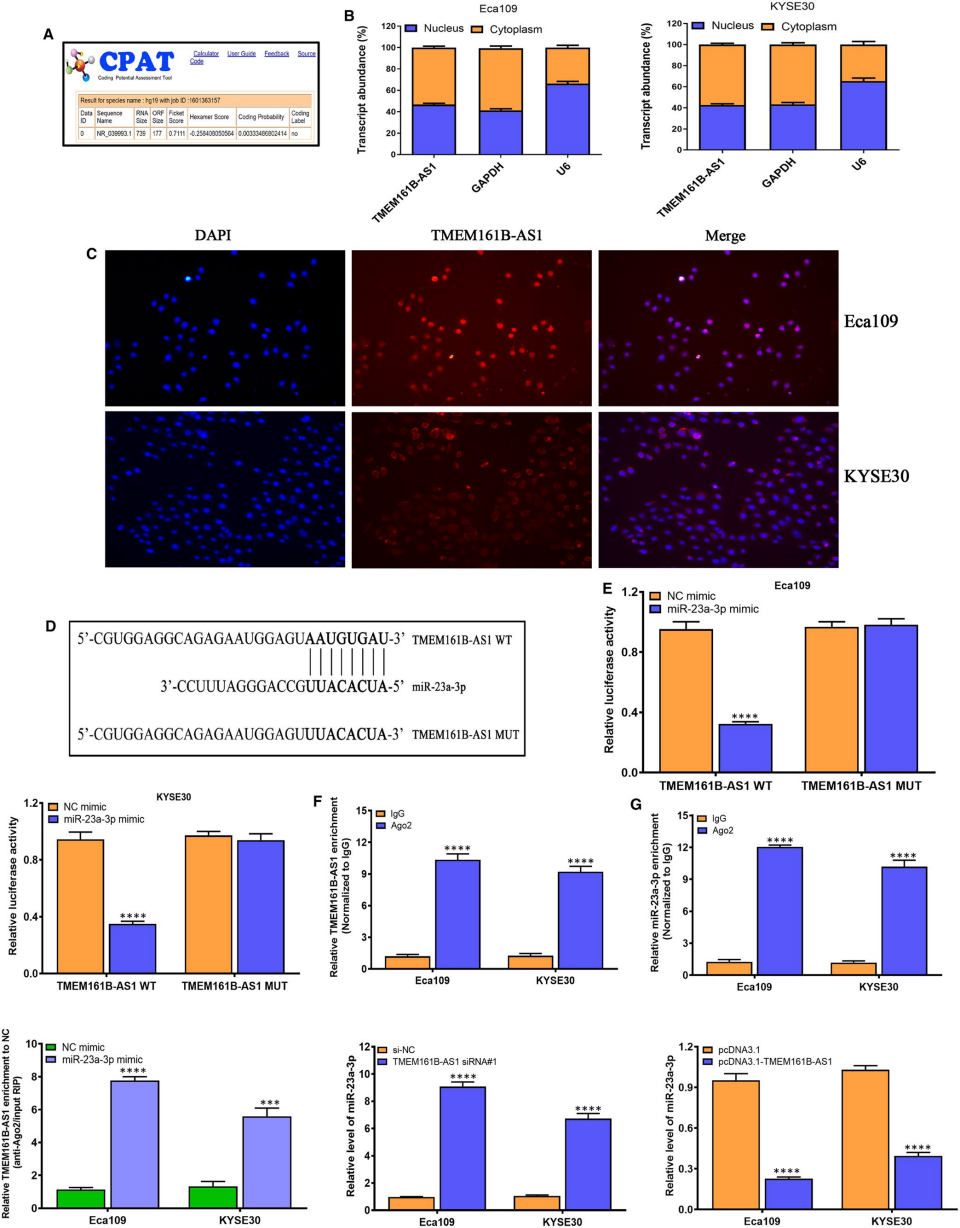
TMEM161B‐AS1 acts as ceRNA by sponging miR‐23a‐3p in ESCC cells. A, CPAT online tool (http://lilab.research.bcm.edu/cpat/) was performed to predict the coding probability of TMEM161B‐AS1. B, Nuclear‐cytoplasmic fractionation was used to determine the subcellular localization of TMEM161B‐AS1 in Eca109 and KYSE30 cells. C, Subcellular localization of TMEM161B‐AS1 in Eca109 and KYSE30 cells investigated by FISH experiment; TMEM161B‐AS1 is labelled by Cy3 (red), and nuclei are stained with DAPI (blue). D, LncBase Experimental v.2 was performed to predict miR‐23a‐3p binding sites in the TMEM161B‐AS1 transcript. E, The dual‐luciferase reporter assay system was conducted to determine the interaction of miR‐23a‐3p with TMEM161B‐AS1 in Eca109 and KYSE30 cells. F, Relative enrichment of TMEM161B‐AS1 in RIP using anti‐Ago2 antibody in Eca109 and KYSE30 cells, and the fold enrichment of TMEM161B‐AS1 normalized to IgG as negative control. G, Relative enrichment of miR‐23a‐3p in RIP using anti‐Ago2 antibody in Eca109 and KYSE30 cells, and the fold enrichment of miR‐23a‐3p normalized to IgG as negative control. H, Relative enrichment of TMEM161B‐AS1 in Eca109 and KYSE30 cells transfected with miR‐23a‐3p mimic or NC mimic. I, qRT‐PCR assay for miR‐23a‐3p level in Eca109 and KYSE30 cells transfected with si‐NC or TMEM161B‐AS1 siRNA#1. J, qRT‐PCR assay for miR‐23a‐3p level in Eca109 and KYSE30 cells transfected with pcDNA3.1 or pcDNA3.1‐TMEM161B‐AS1. ***P < .001 and ****P < .0001, indicating statistical significance

The coding probability of TMEM161B‐AS1 was predicted using online tool CPAT (http://lilab.research.bcm.edu/cpat/), and the data revealed that TMEM161B‐AS1 had no protein‐coding potential (Figure 3A). Subsequently, the subcellular localization of TMEM161B‐AS1 was determined using nuclear‐cytoplasmic fractionation, and we found that TMEM161B‐AS1 was appeared in both cell nuclear and cytoplasm of Eca109 and KYSE30, but mainly appearing in cytoplasm (Figure 3B), which was further verified by FISH experiment in Eca109 and KYSE30 cells (Figure 3C), implying its complex and diversity of function. To predict the underlying targets of TMEM161B‐AS1, LncBase Experimental v.2 was performed to determine the possible binding sites of miRNA in TMEM161B‐AS1 transcript. We found the transcript of TMEM161B‐AS1 had the binding site of miR‐23a‐3p (Figure 3D). To validate this predictive result, we then carried out dual‐luciferase reporter experiment to verify the possible binding of TMEM161B‐AS1 and miR‐23a‐3p in ESCC cells. We found miR‐23a‐3p significantly reduced the luciferase activity in Eca109 and KYSE30 cells transfected with TMEM161B‐AS1 WT vector, but not in TMEM161B‐AS1 MUT vector, suggesting that miR‐23a‐3p is a direct target of TMEM161B‐AS1 (Figure 3E). Further RIP experiment revealed that relative level of TMEM161B‐AS1 and miR‐23a‐3p in anti‐Ago2 antibody group were obviously enhanced compared to IgG group (Figure 3F,G). Furthermore, the relative enrichment of TMEM161B‐AS1 in miR‐23a‐3p mimic group was markedly enhanced, compared with NC mimic group (Figure 3H). Meanwhile, TMEM161B‐AS1 silencing significantly promoted miR‐23a‐3p expression in Eca109 and KYSE30 cells, whereas TMEM161B‐AS1 overexpression dramatically suppressed miR‐23a‐3p expression in Eca109 and KYSE30 cells (Figure 3I,J). These data imply that TMEM161B‐AS1 functions as ceRNA to manipulate miR‐23a‐3p level in ESCC cells.

### 3.4 Enhanced miR‐23a‐3p expression in ESCC tissues and its high expression predicts poor prognosis of ESCC patients

**FIGURE 4:**
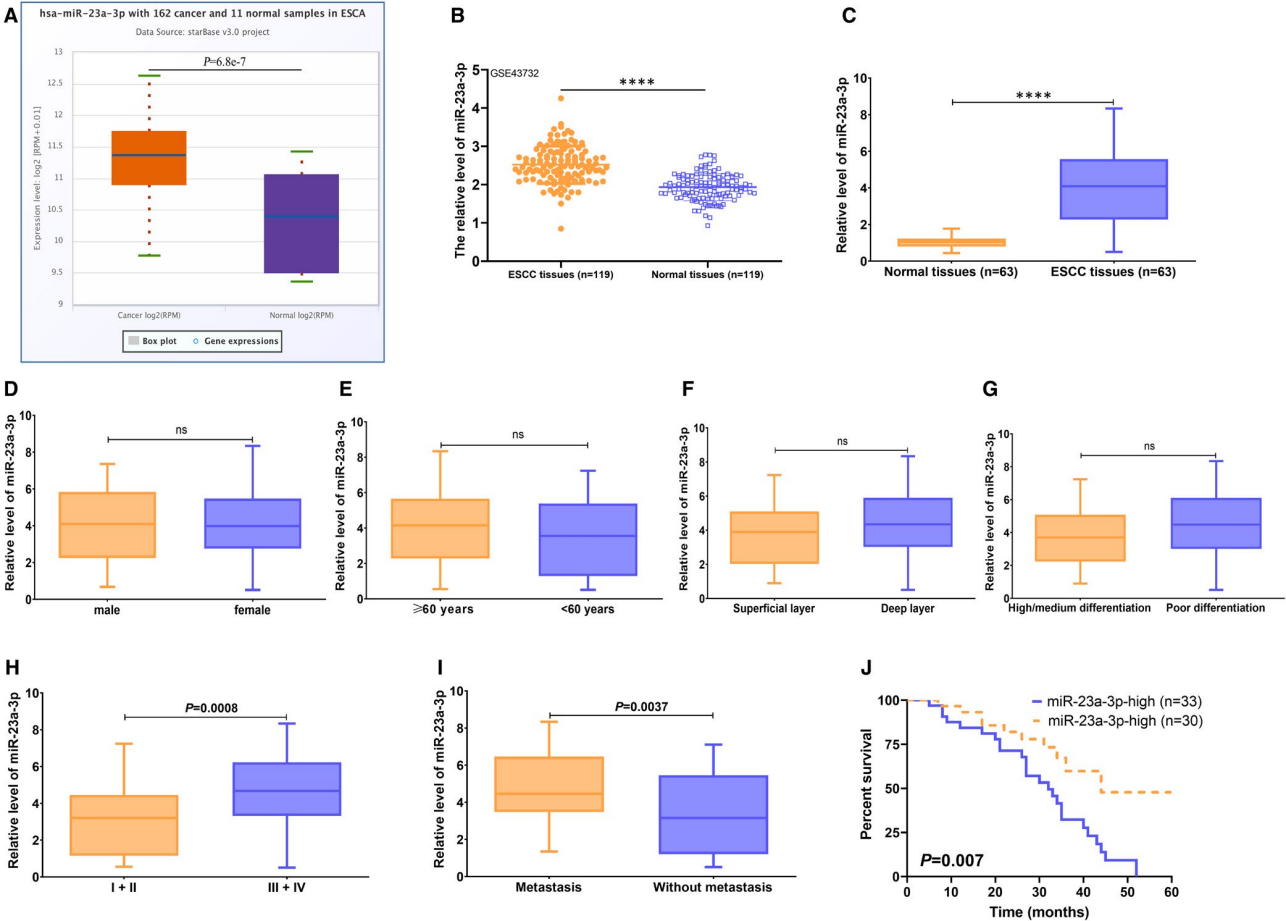
Increased miR‐23a‐3p level in ESCC tissues and its association with the prognosis of ESCC patients. A, StarBase v3.0 online tool was performed to investigate the miR‐23a‐3p expression in 162 cases of ESCA samples and 11 normal samples. B, GEO data set GSE43732 was performed to the expression of miR‐23a‐3p in 119 cases of ESCC samples and paired normal samples. C, qRT‐PCR was used to determine the expression of miR‐23a‐3p in 63 cases of ESCC samples and paired normal samples. D‐I, The expression of miR‐23a‐3p in ESCC samples with different clinicopathological features including gender, age, invasive depth, differentiation degree, TNM stage and lymph node metastasis. J, Log‐rank test was used to determine the association of miR‐23a‐3p expression with the prognosis of ESCC patients. ns indicating no significance. ****P < .0001, indicating statistical significance

To further investigate the expression pattern of miR‐23a‐3p in ESCC tissues, TCGA database, GEO data set and qRT‐PCR were employed to detect its expression and clinic value in ESCC. The data derived from TCGA and GEO data set GSE43732 revealed that ESCA tissues displayed higher miR‐23a‐3p level than normal samples (Figure 4A,B), which was further validated by qRT‐PCR in 63 cases of ESCC tissues and paired normal samples (Figure 4C). Further investigation revealed that the expression of miR‐23a‐3p was not associated with ESCC patients’ gender, age, invasion depth and differentiation degree (Figure 4D‐G), but tightly correlated with TNM stage and lymph node metastasis (Figure 4H,I). Importantly, high miR‐23a‐3p level predicted poor prognosis of ESCC patients (Figure 4J). These data suggest that miR‐23a‐3p may be implicated in ESCC progression and may be new prognostic factor for ESCC patients.

### 3.5 miR‐23a‐3p down‐regulation suppresses the proliferation and invasion of ESCC cells

**FIGURE 5:**
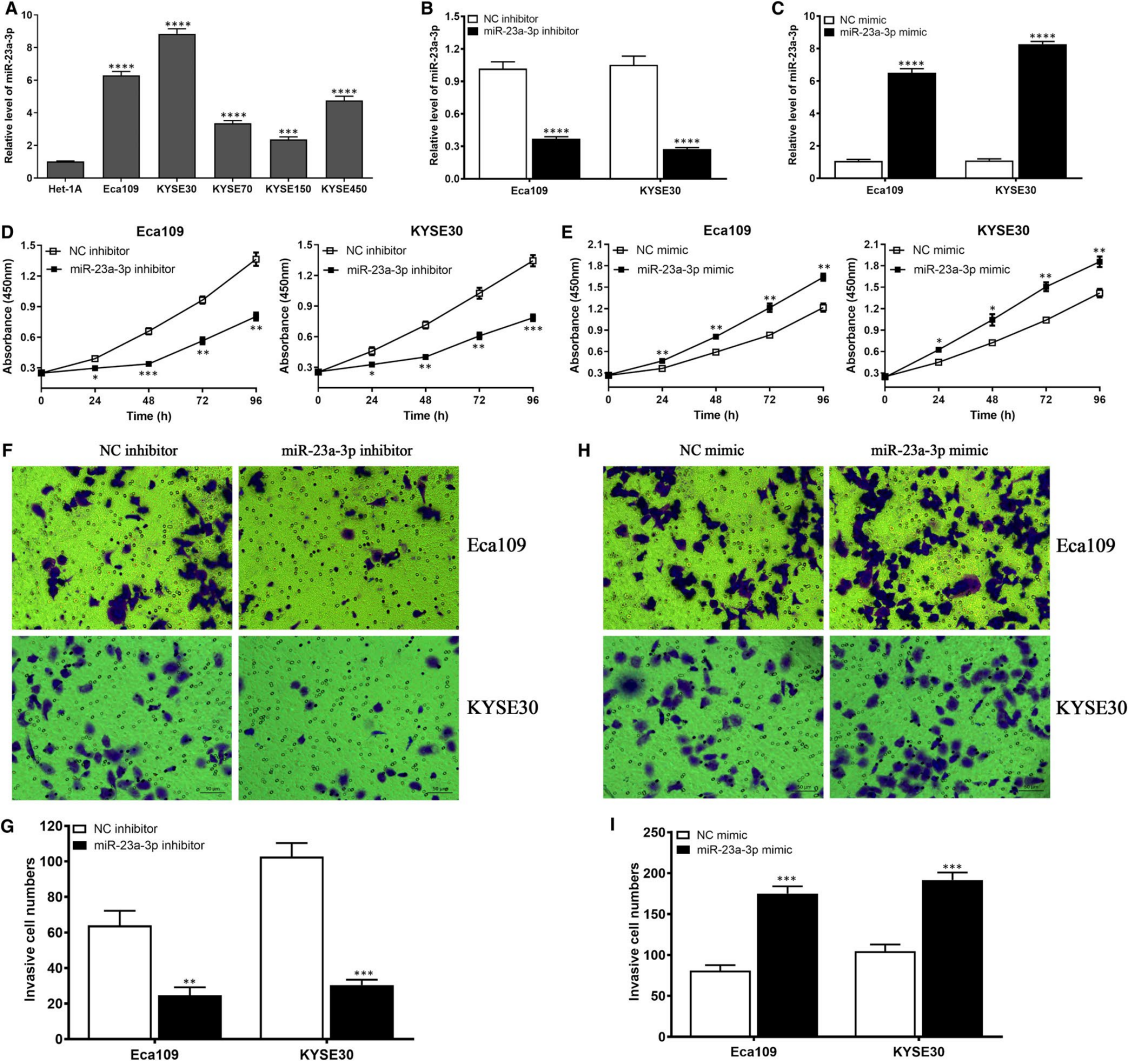
miR‐23a‐3p promotes cell proliferation and invasion in Eca109 and KYSE30 cells. A, The expression of miR‐23a‐3p in a panel of ESCC cells (Eca109, KYSE30, KYSE70, KYSE150 and KYSE450) and normal oesophageal epithelial cell Het‐1A. B, miR‐23a‐3p inhibitor suppressed the expression of miR‐23a‐3p in Eca109 and KYSE30 cells. C, miR‐23a‐3p mimic promoted the expression of miR‐23a‐3p in Eca109 and KYSE30 cells. D, miR‐23a‐3p inhibitor suppressed cell proliferation in Eca109 and KYSE30 cells. E, miR‐23a‐3p mimic enhanced cell proliferation in Eca109 and KYSE30 cells. F and G, miR‐23a‐3p inhibitor suppressed cell invasion in Eca109 and KYSE30 cells, bar = 50 μm. H and I, miR‐23a‐3p mimic promoted cell invasion in Eca109 and KYSE30 cells, bar = 50 μm. *P < .05, **P < .01, ***P < .001 and ****P < .0001, indicating statistical significance

To preliminarily dissect the underlying functions of miR‐23a‐3p in ESCC cells, CCK‐8 kit and Transwell experiments were performed to investigate the effects of miR‐23a‐3p on the proliferation and invasion of ESCC cells. We firstly detected the expression of miR‐23a‐3p in a series of ESCC cells, and the results revealed that ESCC cells exhibited the higher miR‐23a‐3p level than normal oesophageal epithelial cell Het‐1A, in which Eca109 and KYSE30 displayed the relative higher miR‐23a‐3p level (Figure 5A). Besides, miR‐23a‐3p inhibitor significantly suppressed the expression of miR‐23a‐3p in Eca109 and KYSE30 cells (Figure 5B), whereas miR‐23a‐3p mimic markedly promoted the expression of miR‐23a‐3p in Eca109 and KYSE30 cells (Figure 5C). Functionally, miR‐23a‐3p depletion dramatically suppressed the proliferation of Eca109 and KYSE30 cells (Figure 5D); conversely, miR‐23a‐3p overexpression extremely promoted the proliferation of Eca109 and KYSE30 cells (Figure 5E). Transwell experiment demonstrated that miR‐23a‐3p knockdown evidently blocked the invasion of Eca109 and KYSE30 cells (Figure 5F,G), whereas miR‐23a‐3p up‐regulation obviously promoted cell invasion of Eca109 and KYSE30 cells (Figure 5H,I). These data indicate that the suppression of miR‐23a‐3p expression may be a novel therapeutic strategy of ESCC patients.

### 3.6 miR‐23a‐3p promotes the glycolysis of ESCC cells by suppressing HIF1AN

**FIGURE 6:**
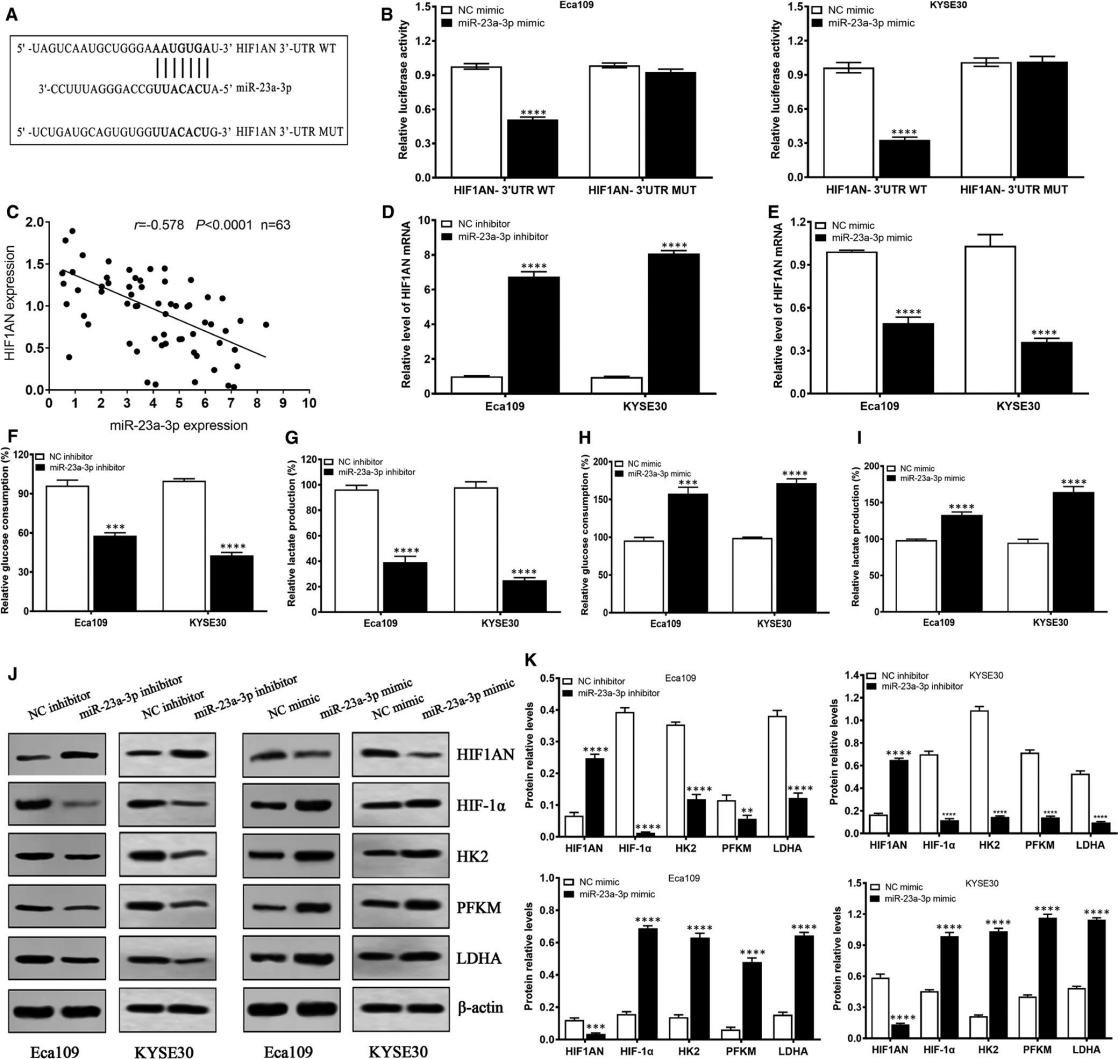
miR‐23a‐6p promotes glycolysis by suppressing HIF1AN expression in ESCC cells. A, TargetScan online tool was performed to predict the possible binding sites of miR‐23a‐3p in HIF1AN 3’‐UTR transcript. B, The dual‐luciferase reporter assay system was conducted to determine the interaction of miR‐23a‐3p with HIF1AN 3’‐UTR in Eca109 and KYSE30 cells. C, Pearson correlation assay was performed to detect the correlation of miR‐23a‐3p expression with HIF1AN expression in 63 cases of ESCC tissues. D, miR‐23a‐3p inhibitor promoted HIF1AN expression in Eca109 and KYSE30 cells. E, miR‐23a‐3p mimic suppressed HIF1AN expression in Eca109 and KYSE30 cells. F, miR‐23a‐3p inhibitor suppressed glucose consumption in Eca109 and KYSE30 cells. G, miR‐23a‐3p inhibitor suppressed lactate production in Eca109 and KYSE30 cells. H, miR‐23a‐3p mimic promoted glucose consumption in Eca109 and KYSE30 cells. G, miR‐23a‐3p mimic improved lactate production in Eca109 and KYSE30 cells. J, The effects of miR‐23a‐3p down‐regulation or up‐regulation on glycolysis‐related proteins HIF1AN, HIF‐1α, HK2, PFKM and LDHA in Eca109 and KYSE30, and β‐actin was used as loading control. K, The relative level of HIF1AN, HIF‐1α, HK2, PFKM and LDHA in Eca109 and KYSE30 after treatment with miR‐23a‐3p inhibitor or mimic. **P < .01, ***P < .001 and ****P < .0001, indicating statistical significance

To unveil the possible molecular mechanisms of miR‐23a‐3p in the glycolysis of ESCC cells, we firstly examined the possible downstream target of miR‐23a‐3p using online tool TargetScan. We found that HIF1AN 3'‐UTR region harboured the binding site of miR‐23a‐3p (Figure 6A). To validate the predictive result, dual‐luciferase reporter experiment revealed miR‐23a‐3p could bind to the 3'‐UTR region in HIF1AN transcript in Eca109 and KYSE30 cells (Figure 6B), suggesting HIF1AN is a direct downstream target gene of miR‐23a‐3p. In addition, miR‐23a‐3p level exhibited negative correlation with HIF1AN level in 63 cases of ESCC samples (Figure 6C). To verify the regulatory correlation of miR‐23a‐3p and HIF1AN, we found that miR‐23a‐3p inhibitor significantly up‐regulated the expression of HIF1AN in Eca109 and KYSE30 cells (Figure 6D), whereas miR‐23a‐3p mimic obviously down‐regulated HIF1AN level in Eca109 and KYSE30 cells (Figure 6E). Importantly, miR‐23a‐3p inhibitor reduced glucose consumption and lactate production in Eca109 and KYSE30 cells (Figure 6F,G), whereas miR‐23a‐3p mimic enhanced glucose consumption and lactate production in Eca109 and KYSE30 cells (Figure 6H,I). To further uncover the possible molecular mechanisms of miR‐23a‐3p in ESCC glycolysis, Western blot was performed to investigate the expressions of glycolysis‐related proteins. We found that miR‐23a‐3p depletion promoted the expression of HIF1AN protein, but suppressed the expressions of HIF‐1α, HK2, PFKM and LDHA proteins in Eca109 and KYSE30 cells (Figure 6J,K); conversely, miR‐23a‐3p up‐regulation restrained the expression of HIF1AN protein, but increased the expressions of HIF‐1α, HK2, PFKM and LDHA proteins in Eca109 and KYSE30 cells (Figure 6J,K). These data suggest that miR‐23a‐3p prompts glycolysis by inhibiting HIF1AN expression and promoting the expressions of glycolysis‐related proteins in ESCC cells.

### 3.7 TMEM161B‐AS1 exerts biological functions in miR‐23a‐3p‐ or HIF1AN‐dependent manner

**FIGURE 7:**
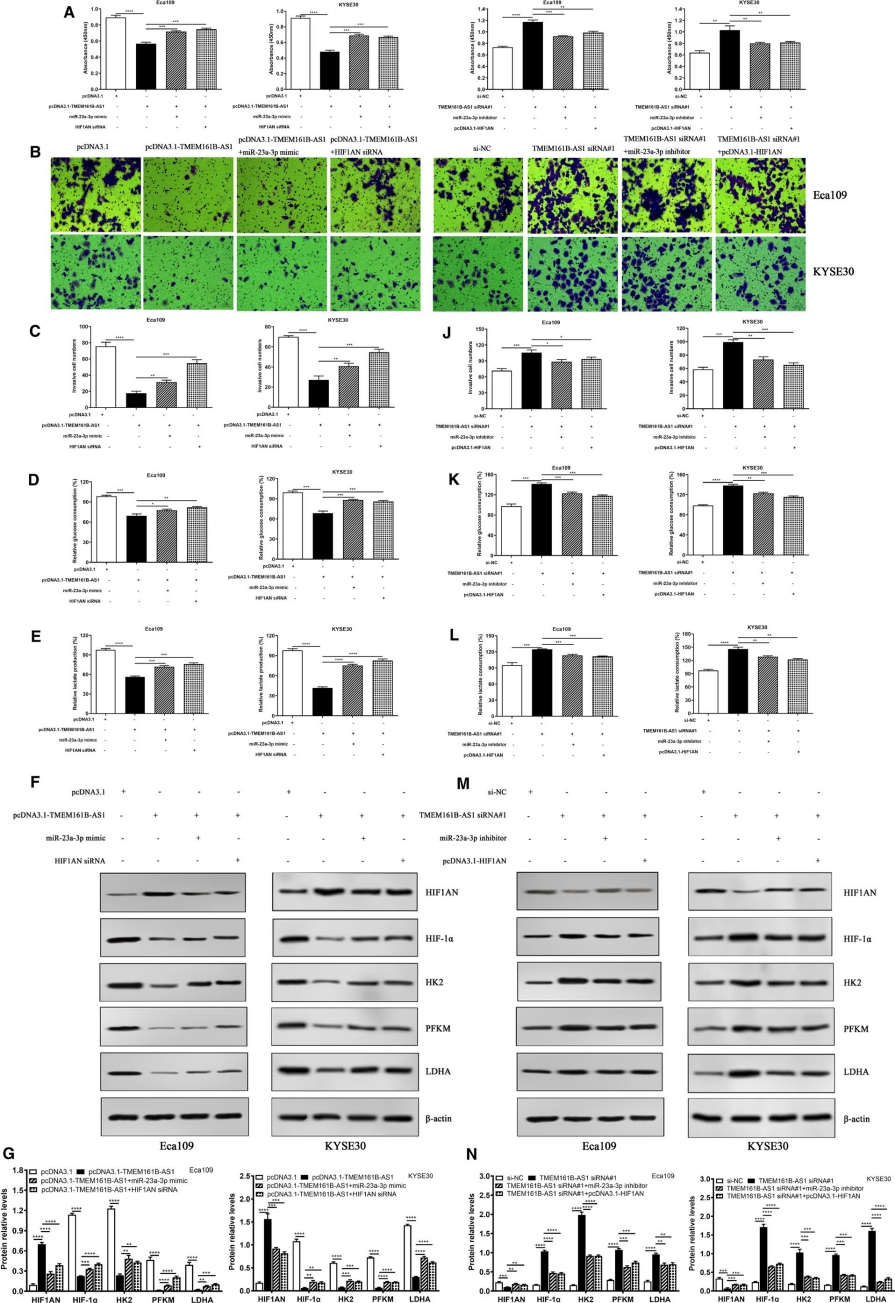
miR‐23a‐3p mimic and HIF1AN siRNA reversed the suppression function of TMEM161B‐AS1 in ESCC cells. A, miR‐23a‐3p mimic and HIF1AN siRNA reversed the proliferation suppression of TMEM161B‐AS1 in Eca109 and KYSE30 cells at 48 h after transfection. B and C, miR‐23a‐3p mimic and HIF1AN siRNA reversed the invasion suppression of TMEM161B‐AS1 in Eca109 and KYSE30 cells at 48 h after transfection, bar = 50 μm. D, miR‐23a‐3p mimic and HIF1AN siRNA reversed the suppression of glucose consumption mediated by TMEM161B‐AS1 in Eca109 and KYSE30 cells at 48 h after transfection. E, miR‐23a‐3p mimic and HIF1AN siRNA reversed the suppression of lactate production evoked by TMEM161B‐AS1 in Eca109 and KYSE30 cells at 48 h after transfection. F and G, miR‐23a‐3p mimic and HIF1AN siRNA reversed the promotion of HIF1AN and suppression of glycolysis‐related proteins HIF‐1α, HK2, PFKM, LDHA regulated by TMEM161B‐AS1 in Eca109 and KYSE30 cells at 48 h after transfection. H, miR‐23a‐3p inhibitor and pcDNA3.1‐HIF1AN reversed the proliferation promotion of TMEM161B‐AS1 siRNA#1 in Eca109 and KYSE30 cells at 48 h after transfection. I and J, miR‐23a‐3p inhibitor and pcDNA3.1‐HIF1AN reversed the invasion promotion of TMEM161B‐AS1 siRNA#1 in Eca109 and KYSE30 cells at 48 h after transfection, bar = 50 μm. K, miR‐23a‐3p inhibitor and pcDNA3.1‐HIF1AN reversed the increase of glucose consumption mediated by TMEM161B‐AS1 siRNA#1 in Eca109 and KYSE30 cells at 48 h after transfection. L, miR‐23a‐3p inhibitor and pcDNA3.1‐HIF1AN reversed the increase of lactate production evoked by TMEM161B‐AS1 siRNA#1 in Eca109 and KYSE30 cells at 48 h after transfection. M and N, miR‐23a‐3p inhibitor and pcDNA3.1‐HIF1AN reversed the down‐regulation of HIF1AN and up‐regulation of glycolysis‐related proteins HIF‐1α, HK2, PFKM and LDHA regulated by TMEM161B‐AS1 in Eca109 and KYSE30 cells at 48 h after transfection. *P < .05, **P < .01, ***P < .001 and ****P < .0001, indicating statistical significance

**FIGURE 8:**
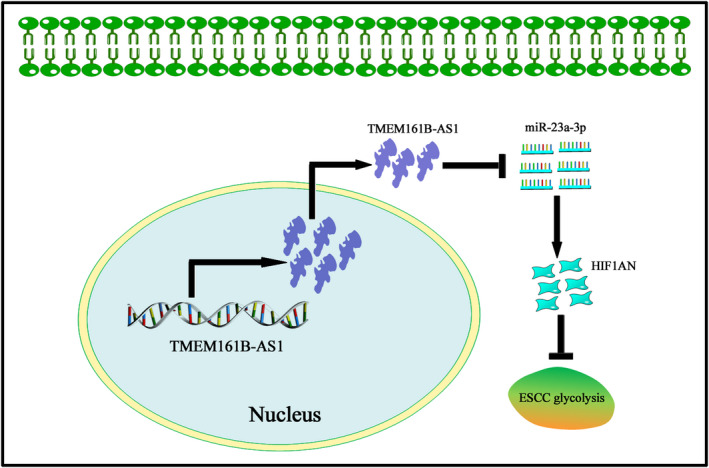
TMEM161B‐AS1 suppresses cell proliferation, invasion and glycolysis by manipulating miR‐23a‐3p/HIF1AN signal axis. TMEM161B‐AS1 exhibits the low level in ESCC tissues and cells, and its overexpression inhibits the expression of miR‐23a‐3p expression in ESCC cells and further results in the up‐regulation of HIF1AN expression in ESCC cells, which further triggers the suppression of ESCC glycolysis

Combined with the data obtained above, we hypothesized that TMEM161B‐AS1 mediated the suppressions of cell proliferation, invasion and glycolysis was dependent on miR‐23a‐3p and HIF1AN level in ESCC cells. To this end, pcDNA3.1‐TMEM161B‐AS1 combined with miR‐23a‐3p mimic or HIF1AN siRNA and TMEM161B‐AS1 siRNA along with miR‐23a‐3p inhibitor or pcDNA3.1‐HIF1AN were applied to rescue experiment. The results revealed that the suppression of cell proliferation, invasion, glucose consumption and lactate production and HFI1AN up‐regulation and the down‐regulations of HIF‐1α, HK2, PFKM and LDHA evoked by TMEM161B‐AS1 overexpression were partly reversed by miR‐23a‐3p mimic or HIF1AN siRNA (Figure 7A‐G). Converse data were obtained when TMEM161B‐AS1 siRNA was combined with miR‐23a‐3p inhibitor or pcDNA3.1‐HIF1AN (Figure 7H‐N). Collectively, these findings suggest that TMEM161B‐AS1 is implicated in the regulation of cell proliferation, invasion and glycolysis by targeting miR‐23a‐3p/HIF1AN signal axis (Figure 8).

## 4 DISCUSSION

Oesophageal cancer is a complex disease that elicits a heavy society burden due to high mortality and poor prognosis.^28^ The dissection of molecular mechanisms of ESCA pathogenesis and the discovery of new molecular targets are drastically critical for ESCA patients. Accumulating evidence has highlighted the pivotal regulatory roles of lncRNAs in ESCC progression, and many lncRNAs may be potential biomarkers for the prognosis of ESCC patients.^29^, ^30^, ^31^, ^32^, ^33^ In this study, we highlighted the roles of TMEM161B‐AS1 in ESCC development, progression and prognosis and demonstrated that TMEM161B‐AS1 blocked cell proliferation, invasion and glycolysis of ESCC cells by absorbing miR‐23a‐3p to enhance HIF1AN expression, further eliciting the inhibition of glycolysis‐related proteins. Our current evidence suggests that TMEM161B‐AS1/miR‐23a‐3p/HIF1AN signal axis may be a new target for the treatment of ESCC patients.

Initially, we found that TMEM161B‐AS1 and HIF1AN was presented as low expression in both ESCC tissues and cells. TMEM161B‐AS1 has been verified to be down‐regulated in high aggressive endometrial cancer cell line,^27^ implying its close association with tumour metastasis. HIF1AN is an asparaginyl hydroxylase, which is implicated in the regulation of HIF signalling.^34^ HIF1AN suppresses the transactivation of HIF via hydroxylation of HIF‐1α at Asn 803 site.^35^ HIF1AN depletion enhances glycolysis by activating HIF,^35^ further targeting glycolysis‐related enzymes to enhance glycolysis and tumour glucose metabolism.^36^, ^37^ Here, we found TMEM161B‐AS1 and HIF1AN expression were both related to TNM stage and lymph node metastasis. It is noteworthy that their low expressions predicted poor prognosis of ESCC patients. More importantly, TMEM161B‐AS1 expression displayed positive correlation with HIF1AN in ESCC tissues, combined with previous reports about HIF1AN function, implying the potential role of TMEM161B‐AS1 in tumour glycolysis. To further test the hypothesis, we performed the corresponding function experiments in ESCC cells, we found that TMEM161B‐AS1 overexpression suppressed cell proliferation, invasion and glycolysis, coupled with increased HIF1AN expression as well as reduced expressions of glycolysis‐related proteins HIF‐1α, HK2, PFKM and LDHA, and converse data were obtained when TMEM161B‐AS1 was depleted. These findings suggest that TMEM161B‐AS1 may be tightly associated with ESCC glycolysis.

Accumulating evidence has demonstrated that lncRNA functions as ceRNA by sponging miRNAs to enhance the expressions of target genes, which will provide new insights into uncharacterized lncRNAs.^38^ To further uncover the regulatory mechanisms of TMEM161B‐AS1 in ESCC cells, we conducted the localization assay for TMEM161B‐AS1 in ESCC cells by nuclear‐cytoplasmic fractionation and FISH experiment. Current data suggested that TMEM161B‐AS1 widely appeared in cell nucleus and cytoplasm of ESCC cells, but mainly in cytoplasm, implying TMEM161B‐AS1 may function as ceRNA in ESCC cells. In general, cytoplasmic lncRNAs usually exert ceRNA function to regulate the expressions of different genes.^39^ Combined with the data of subcellular localization of TMEM161B‐AS1, online tool LncBase Experimental v.2 was used to determine the possible binding sites of miRNA in TMEM161B‐AS1 transcript, and miR‐23a‐3p was verified as a direct target of TMEM161B‐AS1 by dual‐fluorescence reporter assay system and Ago2‐RIP experiment. These data imply that TMEM161B‐AS1 functions as ceRNA by manipulating miR‐23a‐3p level in ESCC cells.

To data, miR‐23a‐3p has been reported to be implicated in the development, progression and prognosis in multiple different tumour types. miR‐23a‐3p exhibited high expression in endoplasmic reticulum–stressed hepatocellular carcinoma (HCC)–derived exosomes, and its high expression predicted poor prognosis of HCC patients; mechanistically, miR‐23a‐3p regulated the expression of PD‐L1 via PTEN‐AKT signalling pathway.^40^ In addition, miR‐23a‐3p functioned as tumour oncogene in renal cell carcinoma, and miR‐23a‐3p silence inhibited the proliferation and mobility in RCC cells by targeting PNRC2.^41^ Another evidence revealed miR‐23a‐3p acted as tumour suppressor, its low expression predicted poor clinical outcome, and miR‐23a‐3p overexpression significantly inhibited the proliferation, invasion and tumorigenicity by targeting adenylate cyclase 1 (ADCY1).^42^ Besides, miR‐23a‐3p was markedly decreased, and might be a potential prognostic indicator in oral squamous cell carcinoma.^43^ The differential expression patterns of miR‐23a‐3p in a variety of tumours prompted us to further unveil its expression status in ESCC tissues and cells and possible molecular mechanisms. Here, we revealed high miR‐23a‐3p expression in ESCC tissues and cells, and its high expression was tightly associated with TNM stage, lymph node metastasis and poor prognosis of ESCC patients. Functionally, miR‐23a‐3p depletion suppressed the proliferation, invasion and glycolysis of ESCC cells, and opposite data were obtained in the present of miR‐23a‐3p mimic. Further mechanistic assay revealed that HIF1AN was a direct downstream target gene of miR‐23a‐3p, and there was a significantly negative correlation between miR‐23a‐3p and HIF1AN expressions in ESCC tissues. Most importantly, miR‐23a‐3p played an important role in ESCC glycolysis by targeting HIF1AN, a glycolysis‐related regulatory protein, which was further implicated in ESCC progression.

On the basis of the information stated above, we hypothesized that TMEM161B‐AS1 mediated the suppressions of cell proliferation, invasion and glycolysis was dependent on miR‐23a‐3p or HIF1AN level in ESCC cells. To this end, pcDNA3.1‐TMEM161B‐AS1 combined with miR‐23a‐3p mimic or HIF1AN siRNA as well as TMEM161B‐AS1 siRNA along with miR‐23a‐3p inhibitor or pcDNA3.1‐HIF1AN were applied to rescue experiment for further elucidation of TMEM161B‐AS1 functions in ESCC cells. Our current data suggest that the suppression of cell proliferation, invasion and glycolysis triggered by TMEM161B‐AS1 overexpression was partly reversed by miR‐23a‐3p mimic or HIF1AN siRNA, and opposite data were obtained when TMEM161B‐AS1 siRNA was combined with miR‐23a‐3p inhibitor or pcDNA3.1‐HIF1AN. These findings indicate that TMEM161B‐AS1 plays a pivotal regulatory role in cell proliferation, invasion and glycolysis by manipulating miR‐23a‐3p/HIF1AN/glycolysis‐related enzyme pathway in ESCC.

In summary, our current data highlight the roles of TMEM161B‐AS1, HIF1AN and miR‐23a‐3p in ESCC development and progression and stress their prognostic values in ESCC patients. Most importantly, TMEM161B‐AS1 suppresses cell proliferation, invasion and glycolysis by absorbing miR‐23a‐3p to promote the expression of HIF1AN, further eliciting the down‐regulation of glycolysis‐related proteins, which eventually results in the suppression of ESCC progression. Our current data support that TMEM161B‐AS1/HIF1AN/miR‐23a‐3p signal axis may be a new target for the treatment of ESCC patients.

## 5 ETHICS APPROVAL AND CONSENT TO PARTICIPATE

This study was reviewed and approved by the Research and Ethics Committee of the Henan Provincial People's Hospital, People's Hospital of Zhengzhou University (Zhengzhou, China). The study was conducted in accordance with the International Ethical Guidelines for Biomedical Research Involving Human Subjects. All patients provided informed consent to participate in the study.

## CONFLICT OF INTERESTS

The authors declare that there are no conflicts of interest.

## AUTHOR CONTRIBUTION

**Zuxuan Shi:** Data curation (lead); Investigation (lead). **Guanghui Li:** Investigation (supporting); Methodology (lead). **Zhen Li:** Investigation (supporting); Writing‐review & editing (supporting). **Junhao Liu:** Investigation (supporting); Validation (supporting); Writing‐review & editing (supporting). **Yu Tang:** Project administration (lead); Supervision (lead); Writing‐original draft (lead).

## Data Availability

The data used to support the findings of this study are available from the corresponding author upon reasonable request.

## References

[1] Alsop BR , Sharma P . Esophageal cancer. Gastroenterol Clin North Am. 2016;45:399‐412. 27546839 10.1016/j.gtc.2016.04.001

[2] Domper Arnal MJ , Ferrández Arenas Á , Lanas AÁ . Esophageal cancer: risk factors, screening and endoscopic treatment in Western and Eastern countries. World J Gastroenterol. 2015;21:7933‐7943. 26185366 10.3748/wjg.v21.i26.7933 PMC4499337

[3] Huang FL , Yu SJ . Esophageal cancer: risk factors, genetic association, and treatment. Asian J Surg. 2018;41:210‐215. 27986415 10.1016/j.asjsur.2016.10.005

[4] Zhang Y . Epidemiology of esophageal cancer. World J Gastroenterol. 2013;19:5598‐5606. 24039351 10.3748/wjg.v19.i34.5598 PMC3769895

[5] Short MW , Burgers KG , Fry VT . Esophageal cancer. Am Fam Physician. 2017;95:22‐28. 28075104

[6] Arnold M , Soerjomataram I , Ferlay J , Forman D . Global incidence of oesophageal cancer by histological subtype in 2012. Gut. 2015;64:381‐387. 25320104 10.1136/gutjnl-2014-308124

[7] Yang H , Liu H , Chen Y , et al. Neoadjuvant chemoradiotherapy followed by surgery versus surgery alone for locally advanced squamous cell carcinoma of the esophagus (NEOCRTEC5010): a phase III multicenter, randomized, open‐label clinical trial. J Clin Oncol. 2018;36:2796‐2803. 30089078 10.1200/JCO.2018.79.1483 PMC6145832

[8] Pickens A , Orringer MB . Geographical distribution and racial disparity in esophageal cancer. Ann Thorac Surg. 2003;76:S1367‐S1369. 14530066 10.1016/s0003-4975(03)01202-5

[9] Siegel RL , Miller KD , Jemal A . Cancer statistics, 2017. CA: Cancer J Clin. 2017;67(1):7–30. 28055103 10.3322/caac.21387

[10] Lu YF , Yu JR , Yang Z , et al. Promoter hypomethylation mediated upregulation of MicroRNA‐10b‐3p targets FOXO3 to promote the progression of esophageal squamous cell carcinoma (ESCC). J Exp Clin Cancer Res. 2018;37:301. 30514328 10.1186/s13046-018-0966-1 PMC6280546

[11] Shao Y , Ye M , Jiang X , et al. Gastric juice long noncoding RNA used as a tumor marker for screening gastric cancer. Cancer. 2014;120:3320‐3328. 24986041 10.1002/cncr.28882

[12] Ponting CP , Oliver PL , Reik W . Evolution and functions of long noncoding RNAs. Cell. 2009;136:629‐641. 19239885 10.1016/j.cell.2009.02.006

[13] Loewen G , Jayawickramarajah J , Zhuo Y , Shan B . Functions of lncRNA HOTAIR in lung cancer. J Hematol Oncol. 2014;7:90. 25491133 10.1186/s13045-014-0090-4 PMC4266198

[14] Ni W , Zhang Y , Zhan Z , et al. A novel lncRNA uc.134 represses hepatocellular carcinoma progression by inhibiting CUL4A‐mediated ubiquitination of LATS1. J Hematol Oncol. 2017;10:91. 28420424 10.1186/s13045-017-0449-4 PMC5395742

[15] Fatica A , Bozzoni I . Long non‐coding RNAs: new players in cell differentiation and development. Nat Rev Genet. 2014;15:7‐21. 24296535 10.1038/nrg3606

[16] Kornienko AE , Guenzl PM , Barlow DP , Pauler FM . Gene regulation by the act of long non‐coding RNA transcription. BMC Biol. 2013;11:59. 23721193 10.1186/1741-7007-11-59 PMC3668284

[17] Nakagawa S , Kageyama Y . Nuclear lncRNAs as epigenetic regulators‐beyond skepticism. Biochem Biophys Acta. 2014;1839:215‐222. 24200874 10.1016/j.bbagrm.2013.10.009

[18] Xu W , Zhou G , Wang H , et al. Circulating lncRNA SNHG11 as a novel biomarker for early diagnosis and prognosis of colorectal cancer. Int J Cancer. 2020;146:2901‐2912. 31633800 10.1002/ijc.32747

[19] Chao Y , Zhou D . lncRNA‐D16366 is a potential biomarker for diagnosis and prognosis of hepatocellular carcinoma. Med Sci Monit. 2019;25:6581‐6586. 31475695 10.12659/MSM.915100 PMC6738002

[20] He A , He S , Peng D , et al. Prognostic value of long non‐coding RNA signatures in bladder cancer. Aging. 2019;11:6237‐6251. 31433789 10.18632/aging.102185 PMC6738399

[21] Chen Y , Bi F , An Y , Yang Q . Identification of pathological grade and prognosis‐associated lncRNA for ovarian cancer. J Cell Biochem. 2019;120:14444‐14454. 31034644 10.1002/jcb.28704

[22] Tang J , Yan T , Bao Y , et al. LncRNA GLCC1 promotes colorectal carcinogenesis and glucose metabolism by stabilizing c‐Myc. Nat Commun. 2019;10:3499. 31375671 10.1038/s41467-019-11447-8 PMC6677832

[23] Zheng X , Han H , Liu GP , et al. LncRNA wires up Hippo and Hedgehog signaling to reprogramme glucose metabolism. EMBO J. 2017;36:3325‐3335. 28963395 10.15252/embj.201797609 PMC5686550

[24] Zheng YL , Li L , Jia YX , et al. LINC01554‐mediated glucose metabolism reprogramming suppresses tumorigenicity in hepatocellular carcinoma via downregulating PKM2 expression and inhibiting Akt/mTOR signaling pathway. Theranostics. 2019;9:796‐810. 30809309 10.7150/thno.28992 PMC6376468

[25] Ma J , Feng J , Zhou X . Long non‐coding RNA HAGLROS regulates lipid metabolism reprogramming in intrahepatic cholangiocarcinoma via the mTOR signaling pathway. Exp Mol Pathol. 2020;115:104466. 32446859 10.1016/j.yexmp.2020.104466

[26] Lin W , Zhou Q , Wang CQ , et al. LncRNAs regulate metabolism in cancer. Int J Biol Sci. 2020;16:1194‐1206. 32174794 10.7150/ijbs.40769 PMC7053319

[27] Dong P , Xiong Y , Yue J , et al. Long noncoding RNA NEAT1 drives aggressive endometrial cancer progression via miR‐361‐regulated networks involving STAT3 and tumor microenvironment‐related genes. J Exp Clin Cancer Res. 2019;38:295. 31287002 10.1186/s13046-019-1306-9 PMC6615218

[28] Di Pardo BJ , Bronson NW , Diggs BS , Thomas CR Jr , Hunter JG , Dolan JP . The global burden of esophageal cancer: a disability‐adjusted life‐year approach. World J Surg. 2016;40:395‐401. 26630937 10.1007/s00268-015-3356-2

[29] Li Z , Qin X , Bian W , et al. Exosomal lncRNA ZFAS1 regulates esophageal squamous cell carcinoma cell proliferation, invasion, migration and apoptosis via microRNA‐124/STAT3 axis. J Exp Clin Cancer Res. 2019;38:477. 31775815 10.1186/s13046-019-1473-8 PMC6882153

[30] Lin C , Zhang S , Wang Y , et al. Functional role of a novel long noncoding RNA TTN‐AS1 in esophageal squamous cell carcinoma progression and metastasis. Clin Cancer Res. 2018;24:486‐498. 29101304 10.1158/1078-0432.CCR-17-1851

[31] Huang L , Wang Y , Chen J , et al. Long noncoding RNA PCAT1, a novel serum‐based biomarker, enhances cell growth by sponging miR‐326 in oesophageal squamous cell carcinoma. Cell Death Dis. 2019;10:513. 31273188 10.1038/s41419-019-1745-4 PMC6609620

[32] Zhao Y , Wang N , Zhang X , Liu H , Yang S . LncRNA ZEB1‐AS1 down‐regulation suppresses the proliferation and invasion by inhibiting ZEB1 expression in oesophageal squamous cell carcinoma. J Cell Mol Med. 2019;23:8206‐8218. 31638344 10.1111/jcmm.14692 PMC6850966

[33] Han GH , Lu KJ , Wang P , Ye J , Ye YY , Huang JX . LncRNA SNHG16 predicts poor prognosis in ESCC and promotes cell proliferation and invasion by regulating Wnt/beta‐catenin signaling pathway. Eur Rev Med Pharmacol Sci. 2018;22:3795‐3803. 29949155 10.26355/eurrev_201806_15262

[34] Zheng X , Linke S , Dias JM , et al. Interaction with factor inhibiting HIF‐1 defines an additional mode of cross‐coupling between the Notch and hypoxia signaling pathways. Proc Natl Acad Sci USA. 2008;105:3368‐3373. 18299578 10.1073/pnas.0711591105 PMC2265116

[35] Zhang N , Fu Z , Linke S , et al. The asparaginyl hydroxylase factor inhibiting HIF‐1alpha is an essential regulator of metabolism. Cell Metab. 2010;11:364‐378. 20399150 10.1016/j.cmet.2010.03.001 PMC2893150

[36] Semenza GL . HIF‐1: upstream and downstream of cancer metabolism. Curr Opin Genet Dev. 2010;20:51‐56. 19942427 10.1016/j.gde.2009.10.009 PMC2822127

[37] Kaelin WG Jr , Ratcliffe PJ . Oxygen sensing by metazoans: the central role of the HIF hydroxylase pathway. Mol Cell. 2008;30:393‐402. 18498744 10.1016/j.molcel.2008.04.009

[38] Thomson DW , Dinger ME . Endogenous microRNA sponges: evidence and controversy. Nat Rev Genet. 2016;17:272‐283. 27040487 10.1038/nrg.2016.20

[39] Cesana M , Cacchiarelli D , Legnini I , et al. A long noncoding RNA controls muscle differentiation by functioning as a competing endogenous RNA. Cell. 2011;147:358‐369. 22000014 10.1016/j.cell.2011.09.028 PMC3234495

[40] Liu J , Fan L , Yu H , et al. Endoplasmic reticulum stress causes liver cancer cells to release exosomal miR‐23a‐3p and up‐regulate programmed death ligand 1 expression in macrophages. Hepatology. 2019;70:241‐258. 30854665 10.1002/hep.30607 PMC6597282

[41] Quan J , Pan X , Li Y , et al. MiR‐23a‐3p acts as an oncogene and potential prognostic biomarker by targeting PNRC2 in RCC. Biomed Pharmacother. 2019;110:656‐666. 30551118 10.1016/j.biopha.2018.11.065

[42] Ma M , Dai J , Tang H , et al. MicroRNA‐23a‐3p inhibits mucosal melanoma growth and progression through targeting adenylate cyclase 1 and attenuating cAMP and MAPK pathways. Theranostics. 2019;9:945‐960. 30867808 10.7150/thno.30516 PMC6401396

[43] Chen F , Qi S , Zhang X , Wu J , Yang X , Wang R . miR‐23a‐3p suppresses cell proliferation in oral squamous cell carcinomas by targeting FGF2 and correlates with a better prognosis: miR‐23a‐3p inhibits OSCC growth by targeting FGF2. Pathol Res Pract. 2019;215:660‐667. 30606659 10.1016/j.prp.2018.12.021

