# Corrigendum

**DOI:** 10.1111/jcmm.17661

**Published:** 2023-02-15

In Shi et al.,^1^ the image for KYSE30 cells in Figure 3C overlapped with Figure 5B in another paper^2^ due to technical error during image preparation. The correct figure is shown below. The authors confirm all results and conclusions of this article remain unchanged.

**FIGURE 3:**
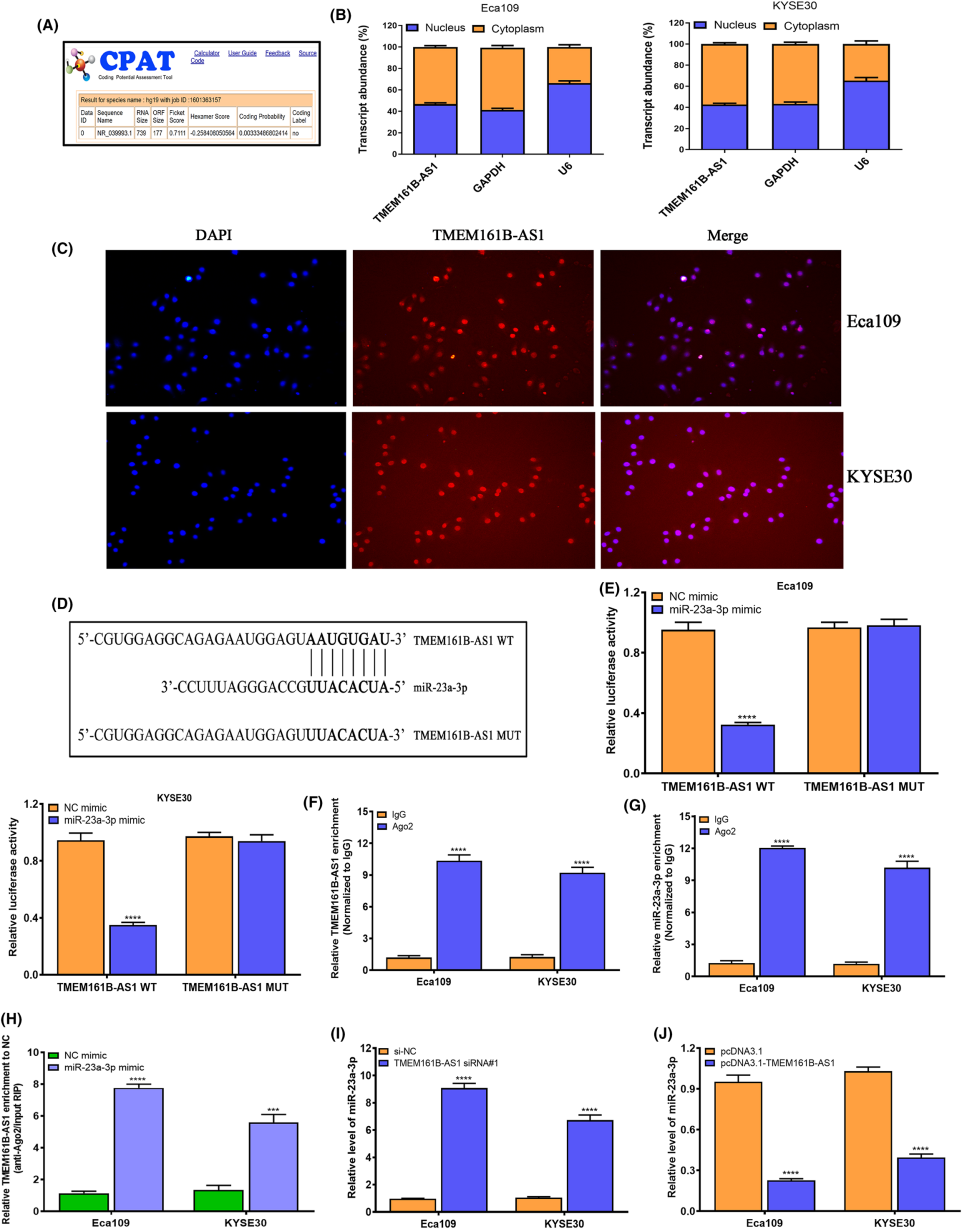
TMEM161B‐AS1 acts as ceRNA by sponging miR‐23a‐3p in ESCC cells. (A) CPAT online tool (http://lilab.research.bcm.edu/cpat/) was performed to predict the coding probability of TMEM161B‐AS1. (B) Nuclear‐cytoplasmic fractionation was used to determine the subcellular localization of TMEM161B‐AS1 in Eca109 and KYSE30 cells. (C) Subcellular localization of TMEM161B‐AS1 in Eca109 and KYSE30 cells investigated by FISH experiment; TMEM161B‐AS1 is labelled by Cy3 (red), and nuclei are stained with DAPI (blue). (D) LncBase Experimental v.2 was performed to predict miR‐23a‐3p binding sites in the TMEM161B‐AS1 transcript. (E) The dual‐luciferase reporter assay system was conducted to determine the interaction of miR‐23a‐3p with TMEM161B‐AS1 in Eca109 and KYSE30 cells. (F) Relative enrichment of TMEM161B‐AS1 in RIP using anti‐Ago2 antibody in Eca109 and KYSE30 cells, and the fold enrichment of TMEM161B‐AS1 normalized to IgG as negative control. (G) Relative enrichment of miR‐23a‐3p in RIP using anti‐Ago2 antibody in Eca109 and KYSE30 cells, and the fold enrichment of miR‐23a‐3p normalized to IgG as negative control. (H) Relative enrichment of TMEM161B‐AS1 in Eca109 and KYSE30 cells transfected with miR‐23a‐3p mimic or NC mimic. (I) qRT‐PCR assay for miR‐23a‐3p level in Eca109 and KYSE30 cells transfected with si‐NC or TMEM161B‐AS1 siRNA#1. (J) qRT‐PCR assay for miR‐23a‐3p level in Eca109 and KYSE30 cells transfected with pcDNA3.1 or pcDNA3.1‐TMEM161B‐AS1. ***p < 0.001 and ****p < 0.0001, indicating statistical significance
